# Medical device development and innovation for rare and pediatric populations: a global landscape overview

**DOI:** 10.1186/s13023-026-04351-0

**Published:** 2026-04-27

**Authors:** Marc Dooms, Daniel O’Connor, Tom Melvin, Friederike Mueller, Vasum Peiris, Katie Pohlson, Durhane Wong-Rieger, Anneliene Hechtelt Jonker

**Affiliations:** 1 University Hospitals Leuven, Leuven, Belgium; 2 International Rare Diseases Research Consortium, Paris, France; 3 ABPI, London, UK; 4 Institute for Clinical Trials, University of Galway, Galway, Ireland; 5 Department of Child and Youth Psychiatry, Psychosomatics and Psychotherapy, Asklepios Hospital Luebben, D-15907 Luebben, Germany; 6 Sanford Health, Sioux Falls, SD USA; 7 Canadian Organization of Rare Disorders, Toronto, Canada; 8 University of Twente, Hallenweg 5, Enschede, 7522 NH The Netherlands

**Keywords:** Orphan devices, Rare diseases, Research and development, Medical technology, Pediatrics

## Abstract

The landscape of orphan therapies has predominantly emphasized drugs and biologics over medical devices for rare diseases. Recent technological advancements, particularly in digital remote monitoring and other innovative technologies, have unveiled new prospects for orphan device development. This shift underscores the potential of these devices in the prevention, diagnosis, monitoring, treatment, and overall enhancement of wellbeing of people living with rare disease. However, despite their significance, only a small percentage of rare diseases currently have approved orphan devices, creating a substantial unmet need for their development. In order to better understand the current orphan device landscape, the International Rare Diseases Research Consortium (IRDiRC) Working Group on Medical Technologies for rare diseases set out to investigate the regulatory landscape, some of the current developments, and the basis of patient engagement in orphan device development. Existing regulatory frameworks vary across regions, offering specific designations and incentives, such as the Humanitarian Device Exemption (HDE) in the United States, Japan’s orphan device designation criteria, and conditional approvals in China. Notably, Europe lacks specific legislation for rare diseases within its medical device regulations, but has developed guidance. Initiatives like the Pediatric Device Consortia Grant Program and System of Hospitals for Innovation in Pediatrics – Medical Devices (SHIP-MD) in the U.S. and the EU4Health co-funded projects in the EU aim to accelerate medical device development, particularly for children and rare diseases. Moreover, patient engagement remains crucial throughout the device development process, emphasizing the need for patient involvement in setting research priorities, study design, execution, and post-approval stages. Furthermore, greater harmonization across regulatory jurisdictions, and greater emphasis on patient engagement may help to facilitate opportunities to address the need for medical devices specifically designed and labelled for rare disease and pediatric populations. Enhancing collaboration among stakeholders, including people living with rare diseases, holds promise for advancing the development of tailored medical devices for rare diseases, ultimately improving patient outcomes and quality of life.

**Supplementary information:**

The online version contains supplementary material available at 10.1186/s13023-026-04351-0.

## Introduction

There are several definitions of a ‘rare disease’ [1, 2]; for the purpose of this paper we use an international operational description of rare disease, which is a medical condition with a specific pattern of clinical signs, symptoms, and findings that affects fewer than or equal to 1 in 2000 persons living in any World Health Organization-defined region of the world [3]. Traditionally the development of therapies for rare diseases has primarily focused on orphan drugs, which are intended specifically for the treatment of rare diseases, with an estimated 5% of rare diseases having a drug available (in the United States or Europe) [4]. Likewise, the development of orphan devices for rare diseases, has been underserved [5]. There are significant differences in the development landscape of orphan drugs and orphan devices, these are summarized in Table 1.

**Table 1:** Key differences in the development landscape for orphan drugs and orphan devices

	Orphan drugs	Orphan devices
Specific legislation	92 countries worldwide [6]	3 countries worldwide
Company involved in marketing the product	Mainly characterized by large companies, with some small and medium enterprises	Mainly characterized by small and medium enterprises with some large companies
Scope of technologies	Characterized by biochemical, metabolic or pharmacological mechanisms of action	Broad spectrum of technologies, encompassing low to high-risk technologies
Costs for health systems	Accumulate over the course of therapy	High initial cost
Use by patients	Relatively simple for patient or caregiver as most delivered by healthcare professionals or simple route of administration	Can be complex and require training prior to use by healthcare professional (for higher risks classes) or patient or caregiver (for lower risk classes)
Iterative development of the technology	Modification to manufacturing process and the label as part of the life cycle	Iterative changes to the technology are common

Orphan devices constitute a very diverse group of products. The definition of a medical device is different across jurisdictions, but generally includes instruments, apparatus, software, implants, or materials used for a medical purpose such as diagnosis, prediction, monitoring, prevention, treatment, or alleviation of a disease [7, 8]. Increasingly so, there are medical devices that contain an element of artificial intelligence or machine learning [9]. Many orphan devices are essential for people living with rare diseases and their caregivers, with medical technologies being used for complex surgery, anesthesia, respiratory support, administration of medications, therapeutic interventions and other services. Medical technologies have the potential to provide a major contribution to the life expectancy and quality of life of people living with rare diseases, for both adult and pediatric populations.

Given the development in the orphan devices field, the International Rare Diseases Research Consortium (IRDiRC) created a Working Group, “Medical Technologies for Rare Diseases,” that aimed to investigate the current regulatory landscape, the current initiatives to support orphan device development, and the need for a patient engagement framework in orphan device development [10]. The topic of orphan devices is being deliberated in many places worldwide, and developing orphan device legislation offers important incentives to encourage the research and development of medical devices for rare diseases. Although similarities exist in the approval procedures in different geographies, these procedures also have key differences, with respect to the definitions used, the availability of regulatory support and guidance, and the financial incentives. This paper, as such, presents a global landscape overview, describing unmet needs, the regulatory landscape, and current initiatives related to orphan medical devices.

### Unmet needs for orphan devices

For the 6000–8000 rare diseases in existence, only a small proportion currently have an approved orphan device. For example, the U.S. FDA listing of humanitarian device exemptions, which apply to high-risk (Class III) devices used for a disease or condition affecting not more than 8,000 patients per year, includes 87 devices since 1997 [11]. While some of these 87 devices can be of value for multiple diseases, there are many diseases that do not have a specific device for their condition or group of conditions. As such, there is a significant unmet need for new devices to be developed [12]. People with rare diseases should be able to expect the same quality and availability of treatment and care as people with a more common disease. However, orphan device developers and innovators experience challenges in translational development that may be an order of magnitude greater than those faced by most device developers, such as the significant cost of clinical investigations, the heterogeneity of people living with rare diseases, the difficulty to recruit patients in clinical trials, and the low return on investment once the device is marketed [13].

Many people with a rare disease have unique needs, and consequently could benefit from devices that are tailored to these needs. A recent needs assessments in the United States aimed to better understand unmet medical device needs for rare diseases, and conducted a survey assessing device needs [14, 15]. This device needs survey highlighted several key findings:

- Most clinicians with expertise in caring for people with rare disease indicated the need for new or improved medical devices for the diagnosis or treatment of rare diseases;
- Existing devices have several limitations in the treatment or diagnosis of rare diseases, and;
- Adapting or repurposing of devices is often not the best solution.

The survey also indicated that current regulatory pathways provide some incentives, but that reimbursement, costs of research, gaining access to Humanitarian Device Exemptions (HDE) [16], institutional review board constraints present significant challenges, and that the pediatric focus for medical devices for rare diseases brings unique challenges, with device needs of pediatric patients with a rare disease being different from those of adults.

In addition, many people with a rare disease are currently dependent on the off-label use of an existing device [17]. Off-label use of a medical device is the use of a medical device in a different way to that described in the instructions for use. In some clinical specialties, for example pediatric cardiology, interventions are dependent on off-label use, when there is no authorized product for the intended use [18]. Off-label use is also a regular occurring practice for drugs and biologics, where they are used off-label for the pharmacological treatment of people living with rare diseases [19, 20]. For some, these medicines continue to be used off-label, while others have now been repurposed as approved orphan drugs [21]. A similar practice can occur in orphan devices through the collection of additional data or real-world evidence, when no other options for the disease are available, and that adapting or repurposing of devices is often not the best solution. It is essential to continue to have currently approved devices available to be used as life-saving orphan devices, regardless of the original intended use.

### Regulatory landscape for orphan devices

To address the challenges for the development of orphan devices, several countries and geographical regions have developed specific orphan device legislation. However, many other countries, such as Australia, Canada, India or Switzerland, do not yet have specific legislation or guidance on orphan devices. Examples of existing programs and processes supported by such legislation include the following:

- The Humanitarian Use Device (HUD) program in the United States, which designates medical devices to treat or diagnose a disease or condition that affects or is manifested in not more than 8,000 individuals in the United States per year [16, 22].
- The Pharmaceutical and Medical Device (PMD) Act in Japan outlines regulations for reviewing orphan devices if they are intended for use in less than 50,000 patients in Japan and for which there is a high medical need [23].
- The Medical Device Administration Regulations (MDAR 2021) in China allows conditional approvals for medical devices use in the treatment of rare or critical diseases where an effective treatment is not available.

Europe’s medical devices are regulated by the Medical Device Regulation (EU) 745/2017 (MDR) [24, 25]. The MDR does not include specific regulations for rare diseases. A guidance document was published in June 2024 concerning the clinical evaluation of orphan devices [26]. This guidance document provides a definition of an orphan devices, and provides input on what is considered sufficient clinical data, and addresses the difficulties in generating this clinical data for orphan medical devices, specifically intended for use in rare diseases, under the MDR. There is currently a proposal of the European Commission to include a definition and legal designation for orphan devices as a revision to the MDR [27].

The criteria and process for orphan device designation is not internationally harmonized and joint applications between Europe, the United States and Japan do not exist. As such, below, we provide an overview of the different features provided for in regulation (Table 2).

**Table 2:** An overview of key features of current legislation for orphan devices

	United States	Japan	European Union	China	Brazil
Legislation for orphan devices	Yes, described in Safe Medical Devices Act	Yes, described in article 77–2 of PMD Act	No	Yes, via Priority Approval Procedure	No, but there are options via the conditional approval system
Start of legislation for orphan devices	1990	1993	-	2021	2022
Organization responsible for granting market access	United States Food and Drug Administration (FDA)	Japan Pharmaceutical and Medical Device Agency and Ministry of Health, Labour and Welfare of Japan	Notified Bodies	China National Medical Products Administration (NMPA)	Brazilian Health Regulatory Agency (ANVISA)
Indication	NA	Serious diseases, including difficult to treat diseases	NA	Rare or critical diseases where an effective treatment is not available	Serious debilitating condition
Guidance and consultation for orphan devices	Yes	Yes	Yes, MDCG guidance and EMA orphan advice pilot	No	No
Reduced fees	Yes	Yes	NA	Yes, for SMEs	Yes, for SMEs
Population-based eligibility criteria	Prevalence: Not more than 8000 individuals in the United States	Incidence: Less than 50,000 individuals in Japan	NA	Unknown, the device needs to be used for a condition included in a list which currently includes 121 diseases	Unknown
Total number of inhabitants	336 M	125 M	450 M	1412 M	214 M
Current number of orphan devices authorized for marketing approval	87	32	NA	Unknown	Unknown

#### United States legislation for medical devices

The United States is one of the countries that has specific legislation for orphan devices that is embedded in the medical device legislation. In the United States, device regulations depend on risk classification: Class I (low risk), Class II (moderate risk), and Class III (high risk). Class I devices generally require manufacturer registration with the FDA and compliance with quality system regulations, but are typically exempt from premarket review [28]. Class II devices usually require FDA clearance through a “510(k)” premarket notification, which may include animal or clinical data if the device differs significantly from a predicate device. Class III devices, which pose the highest risk, require premarket approval (PMA) and must be supported by clinical trial data demonstrating safety and effectiveness for the intended use and patient population [29].

A humanitarian use device (HUD) is a “medical device intended to benefit patients in the treatment or diagnosis of a disease or condition that affects or is manifested in not more than 8,000 individuals in the United States per year” (21 CFR 814.102(a)(5)) [30]. A developer can apply to the U.S. FDA Office of Orphan Products Development (OOPD) to receive a designation as a HUD. For high-risk (Class III) HUDs, market approval is possible through the Humanitarian Device Exemption (HDE) process, as introduced by the Safe Medical Devices Act of 1990 [31]. The HDE allows for a demonstration of probable benefit, rather than the typical evidence standard of safety and effectiveness, and it acknowledges that the development of a device in this context may be impossible, highly impracticable, or unsafe. The HDE application must “contain sufficient information for FDA to determine that the device does not pose an unreasonable or significant risk of illness or injury, and that the probable benefit to health outweighs the risk of injury or illness from its use, taking into account the probable risks and benefits of currently available devices or alternative forms of treatment [32].” In addition, the review timeframe of an HDE application could be shorter than a general pre-market approval (PMA) application. An HDE is only eligible to be sold for profit if the device is intended for use in a pediatric population, and is labelled as such, or occurs in adult patients and does not occur in pediatric patients or occurs in pediatric patients in such numbers that the development of the device for such patients is impossible, highly impracticable, or unsafe [33].

The HDE pathway outlines a number of incentives [34]. Firstly, pragmatic trial designs to demonstrate probable clinical benefit rather than clinical effectiveness can be seen as an incentive. Also, there are no user fees for an HDE application. Furthermore, the regulatory review for HDE application is shorter than for regular PMA applications. Finally, orphan product grants are available for device developers in the rare disease space and direct device funding is available for device developers focused on pediatrics (not limited to rare disease) through the Pediatric Device Consortia grant program, which is funded by the U.S. FDA [35, 36].

#### Japanese legislation for medical devices

In Japan, legislation has been set up that is equal for both orphan drugs and orphan devices for rare diseases. They can be designated based on article 77–2 of the Act on Securing Quality, Efficacy and Safety of Pharmaceuticals, Medical Devices, Regenerative and Cellular Therapy Products, Gene Therapy Products, and Cosmetics [37]. They are designated by the Minister of Health, Labour and Welfare (MHLW) based on the opinion of the Pharmaceutical Affairs and Food Sanitation Council (PAFSC). The pathway for designation for an orphan device and an orphan drug is identical.

There are three designation criteria [38]. In order to be eligible for designation, orphan devices need to intend for use in less than 50,000 patients in Japan. In addition, medical devices should be indicated for the treatment of serious diseases, for which there is a high medical need. There should be no appropriate alternative medical treatment, or the orphan device should be highly efficient compared to existing products. Finally, there should be a possibility of development, a theoretical rationale for the use of the product for the target disease. Further incentives for designated Orphan Devices are the subsidy possibilities via the National Institute of Biomedical Innovation (NIBIO) [39]. Also, there are possibilities to receive guidance and consultation throughout the development process from the Ministry of Health, Labour and Welfare (MHLW), Pharmaceuticals and Medical Devices Agency (PMDA), and NIBIO on research and development of the device. Designated Orphan Devices also receive a priority review possibility, and an extension of the re-examination period. Furthermore, there is a preferential tax treatment for approved devices.

#### European legislation for medical devices

European Union (EU) Directives have applied to medical devices and in vitro diagnostics (IVDs) since the 1990s. In 2017, a new Regulation was published, being the Regulation (EU) 2017/745 (Medical Device Regulation [EU MDR])), governing the safety and performance of devices across their lifetime [40]. The Medical Device Regulation entered into force in May 2021 and is subject to transitional provisions which allow previously approved devices to continue marketing until 2027 or 2028, depending on the risk classification [41].

Unlike in Japan and the United States, no regulatory incentives were included in the MDR for people living with a rare disease. This is different to medicines regulation in Europe which has specific legislation for orphan drugs [1]. Previous public consultation of the European Commission investigated the possibility of developing specific incentives, such as an Orphan Regulation on medical devices and diagnostics [42]. While most responders favored a specific initiative, this was not included in the Regulations. The Medical Device Coordination Group (MDCG), the statutory body responsible for implementation of the Regulations launched an Orphan Device Task Force in October 2021 which has developed guidance and a definition of an orphan medical device [43]. In addition to guidance, the European Medicines Agency has launched a pilot advice program for both developers and Notified Bodies, organizations that are designations to assess the conformity of certain products, to receive scientific advice relating to the clinical evidence requirements for orphan devices.

As a result of the transition from the Directives to the new Regulations, all devices require a re-evaluation to the new rules. In recent years, challenges in accessing assessments by Notified Bodies, in addition to increasing costs of assessments and clinical data requirements have led to a sincere risk of shortages of certain orphan devices [13]. The European Commission acknowledges that additional efforts are needed to address these shortages, and that tailored solutions are needed, in particular for “legacy devices” [44, 45]. There is currently a European Commission proposal to revise the EU MDR, which will allow ‘grandfathering’ (i.e. continued marketing) for legacy orphan devices [27].

In theory, it is possible to apply derogations that avoid the requirement for a notified body assessment on an exceptional basis by means of an application to a national regulatory authority. Following a derogation in one EU Member State it is then possible to apply for an EU-wide derogation, following article 59 of the MDR [46]. This could protect legacy devices at risk of market exit in Europe, however a policy to deploy these derogations in a systematic way for this purpose has not been developed.

### Regulatory initiatives in two other jurisdictions for orphan medical devices: China and Brazil

China’s new Regulation on the Supervising and Administration of Medical Devices took effect on 1 June 2021 [47]. In this regulation a conditional approval is included for urgently needed medical devices used to treat rare or critical diseases where an effective treatment is not available as well as to respond to public health incidents [48]. The conditions for approval will be indicated in the registration certificates. The aim of these incentives is to benefit people living with rare diseases, reducing the difficulties in clinical evaluation and thus promoting the clinical introduction of those devices [49].

In Brazil, access to medical devices for rare diseases is an increasingly important issue for the Brazilian agency ANVISA [50]. All medical devices follow the Registro or Notificação registration pathways based on their risk classification. However, exceptions can apply. Specifically, there’s an avenue to grant people living with a rare disease access to medical devices that are in the developmental stage and lack therapeutic alternatives. Resolution RDC No. 608/2022 introduced the Compassionate Use Program, detailed in the Guidance on the Compassionate Use Program from 04 March 2022 [51]. Despite this, there isn’t a dedicated registration pathway specifically designed for orphan devices or specialized products.

### Other regulatory initiatives that may support orphan medical devices

In the UK, an Innovative Devices Access Pathway (IDAP) was launched in 2023, as a pilot program [52]. IDAP is operated by the Medicines and Healthcare products Regulatory Agency (MHRA), the National Institute for Health and Care Excellence (NICE), Health Technology Wales (HTW) and Scottish Health Technology Group (SHTG). Although not rare disease specific, IDAP aims to take the uncertainty out of developing medical devices, creating an end-to-end pathway to support innovators generate the evidence they need to achieve regulatory approval, Health Technology Assessment (HTA) decisions and access in the UK National Health Service (NHS). HTA in this aspect is a multidisciplinary decision-making progress, informing on decisions on reimbursement, and determining if the new health technology is cost-effective compared to existing standards. IDAP offers an attractive opportunity for those in the rare disease space where there are issues due to the challenges of small population research.

The Harmonization by Doing (HBD) initiative is focused on aligning regulatory standards and practices between the FDA and PMDA via collaboration of governmental, academic, and industrial stakeholders [53–55]. The program aims to facilitate collaboration and harmonization between the regulatory agencies of both countries, and intends to reduce unnecessary differences in regulatory requirements, streamline processes, and promote mutual recognition of each other’s standards for approving pharmaceuticals and medical devices. By harmonizing certain aspects of regulations, the program seeks to enable smoother and more efficient access to safe and effective medical products in both countries. Furthermore, the “Harmonization by Doing for Children” (HBD-for-Children) working group works as a global collaborative activity to focus on promoting the development of pediatric medical devices [56].

The goal is to enhance patient access to innovative medical devices while maintaining stringent safety and efficacy standards. The program often involves joint discussions, information exchange, and shared initiatives between the regulatory bodies to align their approaches and foster a more cohesive regulatory environment for pharmaceuticals and medical devices.

#### Custom made devices

In specific cases, technology developers can develop medical devices for specific people living with a rare disease, defined as a custom-made device, specifically defined in the medical device legislation. A custom-made device is a one-of-a-kind device designed for an immediate need and for which the need is not likely to reoccur, as such requiring the clinician, developer and potentially even the patient to work together to design the device for that occasion. Both the FDA and European MDR have specific regulations set out for the development of these custom-made devices to address their individual condition and needs [57, 58]. Japan does not have a custom-made device regulation. Examples of custom-made devices in an orphan context are 3D printed protheses, for example for rare bone disorders.

### United States initiatives to support orphan device research and development

In addition to the regulatory incentives, there are a number of initiatives that are of high interest in the landscape of orphan device development. Firstly, the United States has established the Pediatric Device Consortia Program, following passage of the Pediatric Medical Device Safety and Improvement Act of 2007 [59]. These consortia aim to address the unique challenges and needs associated with the development and approval of medical devices for pediatric patients living with a rare disease. Furthermore, they are focused on mitigating the challenges across the total product lifecycle, in addition to providing advice on regulatory concerns. This program also specifically aims to facilitate the development of medical devices for this underrepresented population.

The FDA Pediatric Device Consortia are consortia that bring together various stakeholders, including clinicians, researchers, industry representatives, and regulatory experts. These consortia work to accelerate the development and commercialization of medical devices for the pediatric population by providing expertise, resources, and support throughout the device development process. Specifically, they offer technical assistance and expertise, funding support, regulatory guidance, networking and collaboration, and educational resources. While these consortia are aimed at all pediatric diseases, not just rare pediatric diseases, many devices for pediatric patients living with a rare disease are used in the treatment of rare diseases. Currently five consortia are supported under this grant. As part of the 2018 grant cycle, the Pediatric Device Consortia hosted the Pediatric Device Innovators Forum (PDIF), a recurring collaborative educational experience designed to connect, to foster synergy among innovators across the technology development ecosystem who are interested in pediatric medical device development [60]. The European Commission has followed the example of the FDA Pediatric Device Consortia, and has funded the first three consortia in 2024 [61], DeCODe, i4KIDS-4RARE, and OrphaDev4Kids [62–64].

In addition, the System of Hospitals for Innovation in Pediatrics – Medical Devices (SHIP-MD) a strategic framework initially proposed by FDA’s Center for Devices and Radiological Health (CDRH), is a collaboration between the public and private sectors. Stakeholders from U.S. government entities, academic investigators, pediatric medical society representatives, pediatric patient advocates to medical device industry representatives, pediatric health system leaders and financing and reimbursement experts have refined and vetted the framework during phase 1 and have initiated phase 2 (with a minimal alteration to the name: System of Health Innovation for Pediatrics – Medical Devices), with the goal of developing an operational plan [65, 66]. The initiative is aimed at innovating the pediatric medical device ecosystem, streamlining developmental processes, and incentivizing investment and innovation in the pediatric medical device market. A foundational aspect of SHIP-MD is a sustainable, dynamic, and accessible evidence generation infrastructure that may accelerate the development of medical devices designed, evaluated and labelled for pediatrics. Ultimately, successful launch and sustainability of SHIP-MD may improve healthcare for pediatric and rare disease populations.

### European Commission initiatives to support orphan device research and development

In the EU, the Coordination of Research and Evidence for Medical Device (CORE-MD) project was a Horizon-funded initiative to improve the methods for clinical investigation and evaluation of high-risk medical devices. It included a specific work package focused on medical devices for children (0–18 years old) and this group has prepared European expert recommendations on the clinical investigation and evaluation of high-risk medical devices for children [67]. Another relevant initiative is the European Paediatric Translational Research Infrastructure (EPTRI) project which brings together hundreds of research units to support the development of medicines and medical devices for children [63, 68]. Furthermore, there is the Pediatric Innovation Hub initiative, which focuses in bringing innovation to children, and focuses on overcoming barriers in pediatric device development [62, 69].

### Patient engagement in orphan device development

Patient engagement in all aspects of rare disease research continues to gain acceptance and implementation. Specifically, in different legislations for (orphan) medical device development, the role of people living with a rare disease or users in the development is emphasized [70]. Unfortunately, most orphan devices have been developed for patients and their caregivers without their input, from the earliest stages of development onwards. This has in some circumstances where the development of devices were not optimized for the needs of people living with a rare disease or they failed to be beneficial to people living with a rare disease. Moreover, as many people living with a rare disease are children, people of different age groups may regard, adopt and accept devices differently, and not everyone accepts a specific device as the solution for their unmet need [71]. As such, in addition to engagement of caregivers of children across the age spectrum, different groups of people living with a rare disease need to be consulted in order to have solutions that address their different needs.

**Fig. 1:**
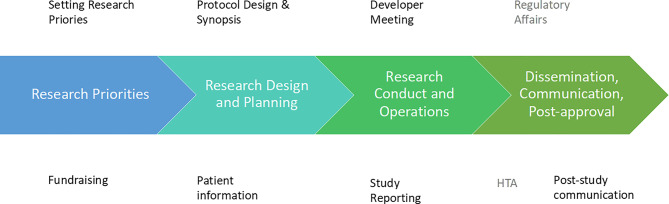
Options for patient engagement in orphan device development. Figure is adapted from Geissler, Ryll, Leto, Uhlenhopp. DOI: 10.1177/2168479017706405

In addition, the highly technological development of devices and the complicated regulatory framework for their approval requires a certain expert level for successful engagement of people living with a rare disease in device research and development. Also, to the best of our knowledge, few practice guidelines have been developed to incorporate this into development practice, that are truly orphan device specific, but are based on overall patient centeredness in rare diseases [72, 73]. Examples of collaboration in specific diseases in the development of orphan devices have shown the benefit of collaboration for different elements, such as arm support for people with Duchenne, or tools to detect rare epilepsy attacks [74–77]. Consequently, the Working Group discussed options for engagement of people living with a rare disease in the development of orphan devices (Fig. 1), based on existing frameworks [72, 73, 78].

The options for engagement can be divided into four phases, similar to medicinal product development [78]. First, people living with a rare disease or their caregivers can assist in setting the research priorities, based on the patient’s need for devices and in some cases can assist in fundraising. It is essential that different stakeholders, such as the engineers, designers, healthcare professionals, people living with a rare disease, and caregivers really come together to engage in constructive debate, so that the development priorities are clear, and reflected in the design input of a new orphan device. This provides opportunities for mutual learning and effective collaboration. In the second phase, the research design and planning phase, people living with a rare disease or their caregivers, can assist in reviewing protocols for clinical studies and clinical endpoints, which allows to better understand the benefits most important to people living with a rare disease and what risks and burdens people living with a rare disease may or may not be willing to tolerate, for example how many visits to hospitals, or which side effects are tolerable. Importantly, devices are often an adjunct to medicinal treatment, either directly to improve the accessibility or impact of the medicine, or as a supplementary “treatment.” To demonstrate the “unique” added value of the device may be very challenging and clinical studies need to be appropriately designed to “tease out” these effects, especially if the device is perceived as contributing to administration and adherence, therefore improving quality of life, but not necessarily having a direct impact on improved clinical outcomes.

People living with a rare disease or their caregivers can also contribute to creating information for other patients as research subjects, for later clinical studies. In the third phase, the execution of research, they can meet regularly with the study sponsor, development team and clinical specialists to discuss if the (preliminary) findings are in line with what was expected or desired. As such they can assist the developer in rejecting those that do not work and improving those that work. In recognizing the diversity of people living with a specific disease or condition, engagement during this execution stage may help surface those patient cohorts who could benefit from the medical device, those who may not, and even those who may experience adverse effects. Finally, in the last phase working towards approval, two regulatory systems (the United States and Japan) [40] have introduced guidance documents (in the United States) [41] and a working group (in Japan) [79, 80] for patient engagement. People living with a rare disease and their caregivers are essential to help in the communication and dissemination of new orphan devices. Finally, reporting of adverse events is an essential part of safety oversight and an important patient role in the oversight of orphan devices and in the materiovigilance period after commercialization of the device. Materiovigilance studies events that arise as a result from medical device usage, such as adverse events [81]. The implementation of patient data platforms to collect data in real-world usage is deemed essential for allowing monitored (conditional) access to some medical devices, contingent on reassessment with real-world evidence over a period of time, analyzed, and used to update knowledge and protocols.

## Conclusion

The IRDiRC Working Group Medical Technology for Rare Diseases investigated the current landscape for orphan devices. The topic of orphan devices is currently developing in many places worldwide, the initiatives and support that we have discussed in this paper are to be welcomed. Developing orphan device legislation offers important incentives to encourage the research and development of medical devices for rare diseases. Although similarities exist in the approval procedures in different geographies, these procedures also have key differences with respect to the definitions used, the availability of regulatory support and guidance, and the financial incentives. In addition to regulatory incentives, there are also a variety of different initiatives to support orphan device development.

In order to deliver the maximum benefit to children and adults with rare diseases we should strive towards a harmonized set of rules, definitions and processes in order to maximize device availability. A greater alignment of clinical evidence infrastructure would facilitate a faster development and deployment of new technologies. Public funding support for post-market registries will set a basis to further develop and deploy these orphan devices, while ensuring that safety concerns are identified as early as possible.

Furthermore, we recommend that the research and development of orphan devices involve people living with rare diseases and their caregivers in identifying unmet needs. Including them at every stage of the process can help streamline development and lead to more effective outcomes. Diverse representation is essential to ensure that multiple perspectives are considered and that the resulting device meets the broad needs of the rare disease community.

## Electronic supplementary material

Below is the link to the electronic supplementary material.

Supplementary material 1

## Data Availability

N/A.

## Abbreviations

ANVISA: Agência nacional de vigilância sanitaria
BARDA: Biomedical advanced research and development authority
CORE-MD: Coordinating research and evidence for medical devices
FDA: U.S. Food and Drug Administration
EEG: Electroencephalogram
EPTRI: European pediatric translational research infrastructure
HBD: Harmonization by doing
HDE: Humanitarian device exemption
HTA: Health technology assessment
HUD: Humanitarian use device
IRDiRC: International rare disease research consortium
IVD: In vitro diagnostic
IVDR: In vitro diagnostic regulations
MDAR: Medical device administration regulation
MDCG: Medical device coordination group
NHS: National health service
MDR: Medical device regulation
MHLW: Japan ministry of health, labour and welfare
NIBIO: National institute of biomedical innovation
NMPA: National medical products administration
OOPD: U.S. FDA office of orphan products development
PAFC: Pharmaceutical affairs and food sanitation council
PDIF: Pediatric device innovators forum
PMA: Pre-market approval
PMDA: Japan pharmaceuticals and medical devices agency
SHIP-MD: System of hospitals for innovation in pediatrics medical device

## Declarations

### Disclaimer

The views expressed in this article are the personal views of the author(s) and may not be understood as being made on behalf of their employ-er(s)/organization(s).
