# Supplementary material for: Medical device development and innovation for rare and pediatric populations: a global landscape overview
Source: Orphanet J Rare Dis. 2026 Apr 27;21:224. doi: 10.1186/s13023-026-04351-0 (PMC13276941; doi:10.1186/s13023-026-04351-0)
Supplement: Supplementary file 1 — Supplementary material 1 [file 13023_2026_4351_MOESM1_ESM.docx]

**Supplementary information: Information Search Strategy**

**1. Literature Search Strategy**

**Databases Searched**

The literature search was conducted in the following databases:

- PubMed: ("rare disease*" OR "orphan disease*" OR "rare condition*" OR "orphan condition*" OR "rare disorder*" OR "orphan disorder*" OR "low prevalence disease*" OR "neglected disease*")AND ("medical device*" OR "biomedical device*" OR "health technology*"OR "diagnostic device*" OR "implantable device*" OR "assistive device*" OR "therapeutic device*") AND (regulation OR regulatory OR legislation OR policy OR policies OR framework* OR approval OR authorization OR governance OR "market approval" OR "premarket approval" OR "regulatory pathway*" OR oversight OR compliance)
- Google Scholar: Orphan devices AND regulation
- Grey literature of regulatory documents

The search covered documents published until June 2025, when the final search was conducted.

**2. Eligibility Criteria**

Studies were included based on the following criteria.

| Criterion | Inclusion | Exclusion |
| --- | --- | --- |
| Publication type | Peer-reviewed articles, grey literature articles. | Editorials, letters, conference abstracts |
| Population | Rare diseases, pediatric rare diseases | Studies on unrelated populations |
| Topic relevance | Directly addressing regulation around devices for rare diseases. | Peripheral or unrelated topics |

**3. Study Selection Process**

The study selection process involved the following steps:

1. Identification of records through database searches
2. Screening of titles and abstracts for relevance
3. Full-text review of potentially eligible articles
4. Expert consultation and working group discussion
5. Final inclusion of studies relevant to the narrative synthesis

**4. Expert Consultation and Working Group Discussions**

To complement the literature search, the topic and emerging themes were discussed within an international working group consisting of 21 experts in orphan medical devices. The group included researchers, regulators, and clinicians from 20 institutions across 10 countries.

Working group meetings were held between March 2022 and June 2025. During these meetings, participants discussed key developments in the field, identified emerging concepts, and suggested relevant literature.

These discussions were used to:

- refine the scope of the review,
- identify additional key publications not captured in the database search, and
- contextualize the interpretation of findings.

Literature identified through expert recommendation was subsequently screened using the same eligibility criteria applied to database search results.
